# Developing an Educational Website for Women With Endometriosis-Associated Dyspareunia: Usability and Stigma Analysis

**DOI:** 10.2196/31317

**Published:** 2022-03-03

**Authors:** Abdul-Fatawu Abdulai, A Fuchsia Howard, Paul J Yong, Heather Noga, Gurkiran Parmar, Leanne M Currie

**Affiliations:** 1 The School of Nursing University of British Columbia Vancouver, BC Canada; 2 Department of Obstetrics & Gynaecology University of British Columbia Vancouver, BC Canada; 3 Women Health Research Institute British Columbia Women's Hospital & Health Center Vancouver, BC Canada

**Keywords:** endometriosis, sexual pain, dyspareunia, usability testing, think-aloud, stigma, web sites, digital health, informatics

## Abstract

**Background:**

Endometriosis is a chronic condition that affects approximately 10% of women worldwide. Despite its wide prevalence, knowledge of endometriosis symptoms, such as pelvic pain, and treatments remains relatively low. This not only leads to a trivialization of symptoms and delayed diagnosis but also fuels myths and misconceptions about pain symptoms. At the same time, the use of web-based platforms for information seeking is particularly common among people with conditions that are perceived as stigmatizing and difficult to discuss. The *Sex, Pain, and Endometriosis* website is an educational resource designed to provide evidence-based information on endometriosis and sexual pain to help people understand the condition, feel empowered, dispel myths, and destigmatize endometriosis-associated sexual pain.

**Objective:**

The study objective is to evaluate the usability of the website and assess for destigmatizing properties of sexual health–related web-based resources.

**Methods:**

We conducted a usability analysis by using a think-aloud observation, a postsystem usability questionnaire, and follow-up interviews with 12 women with endometriosis. The think-aloud data were analyzed using the framework by Kushniruk and Patel for analyzing usability video data, the questionnaire data were analyzed using descriptive statistics, and the follow-up interviews were analyzed using simple content analysis. We conducted a usability assessment by deductively analyzing the interview data via a trauma-informed care framework and a content analysis approach.

**Results:**

Through usability analysis, we found the website to be simple, uncluttered, satisfying, and easy to use. However, 30 minor usability problems related to navigation; website response; the comprehension of graphics, icons, and tabs; the understanding of content; and mismatch between the website and users’ expectations were reported. In our stigma analysis, we found the web content to be nonstigmatizing. The participants suggested ways in which websites could be designed to address stigma, including ensuring privacy, anonymity, inclusiveness, and factual and nonjudgmental content, as well as providing opportunities for web-based engagement.

**Conclusions:**

Overall, the participants found the website to be useful, easy to use, and satisfying. The usability problems identified were largely minor and informed the website redesign process. In the context of the limited literature on stigma and website design, this paper offers useful strategies on how sexual health–related websites can be designed to be acceptable and less stigmatizing to individuals with sensitive health issues.

## Introduction

### Background

Endometriosis is a chronic condition in which endometrial-like tissue is present outside the uterus, typically in the pelvic cavity [1]. Although the actual prevalence of endometriosis may be difficult to quantify, it is estimated that the disease affects approximately 1 out of every 10 women worldwide [2,3]. Women with this disorder tend to experience a variety of symptoms, including sexual pain, menstrual pain, chronic pelvic pain, and infertility [4]. Although pelvic pain is the most common symptom of endometriosis, 50% of women also experience endometriosis-associated dyspareunia—pain experienced during or after penetrative vaginal intercourse [5,6]. Endometriosis-associated dyspareunia affects multiple aspects of life, leading to absenteeism from work, poor interpersonal relationships, and impaired social functioning, and has a profound negative impact on quality of life [7].

Despite the negative effect of sexual pain on the life of people with endometriosis, many patients and health care providers do not take this symptom seriously, whereas others neglect pain symptoms or are unaware of the link between painful sex and endometriosis [8]. Even if the health care provider and patient are both knowledgeable about dyspareunia, the private nature of sexual pain may inhibit patient–provider discussions on the topic [9]. Owing to fear of social stigma, some patients with dyspareunia may also feel reluctant to visit or disclose their sexual pain experiences to health care providers [9]. Indeed, stigma has been identified as a significant barrier to the uptake of sexual health–related interventions, including management of endometriosis-associated dyspareunia [7].

Given the potentially stigmatizing nature of sexual pain, websites have been identified as complementary tools for disseminating patient information concerning endometriosis and its related symptoms [10]. With the internet becoming a major source of health information, people can explore sensitive and intimate health topics such as sexual pain in a private setting. Available evidence suggests that the use of web-based platforms for information seeking is particularly common among people with conditions or symptoms that are perceived as stigmatizing and difficult to discuss [11,12]. Web-based information is particularly important as it may help patients understand the complex relationship between endometriosis-associated pelvic pain and painful sex and improve patients’ access and adherence to recommended treatments while maintaining anonymity [8]. The advantages associated with access to web-based information on sensitive health topics present an opportunity to develop a web-based educational resource for people with endometriosis-associated dyspareunia. In response to this need, we developed a patient-centered educational website to provide evidence-based information on endometriosis-associated dyspareunia, which became the *Sex, Pain, and Endometriosis* website.

### *Sex, Pain, and Endometriosis* Website Development

**Figure 1:**
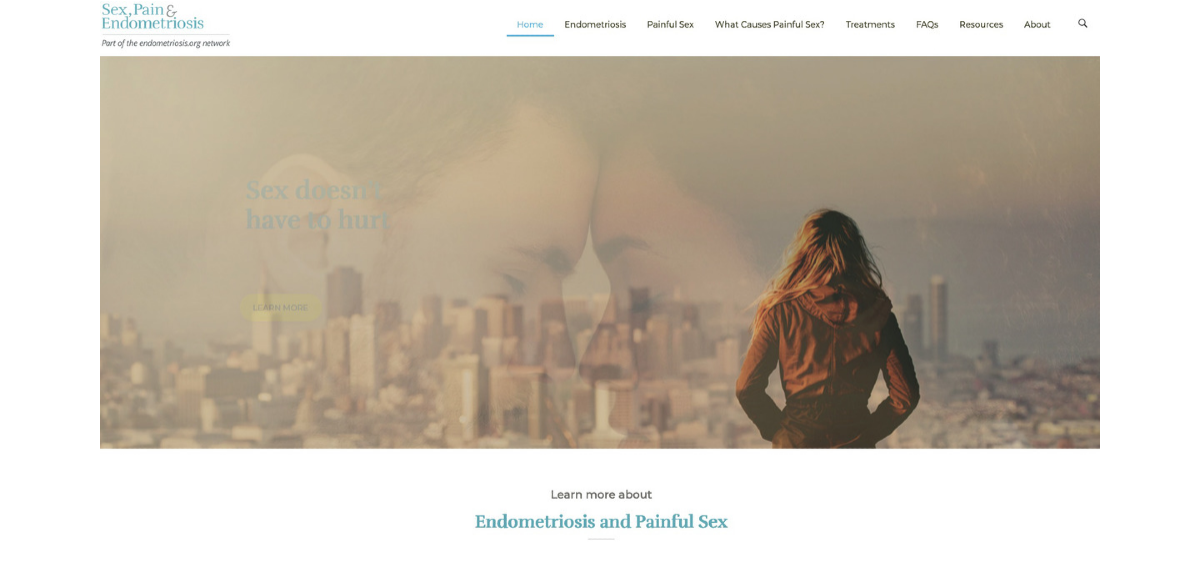
Landing page of the website.

**Figure 2:**
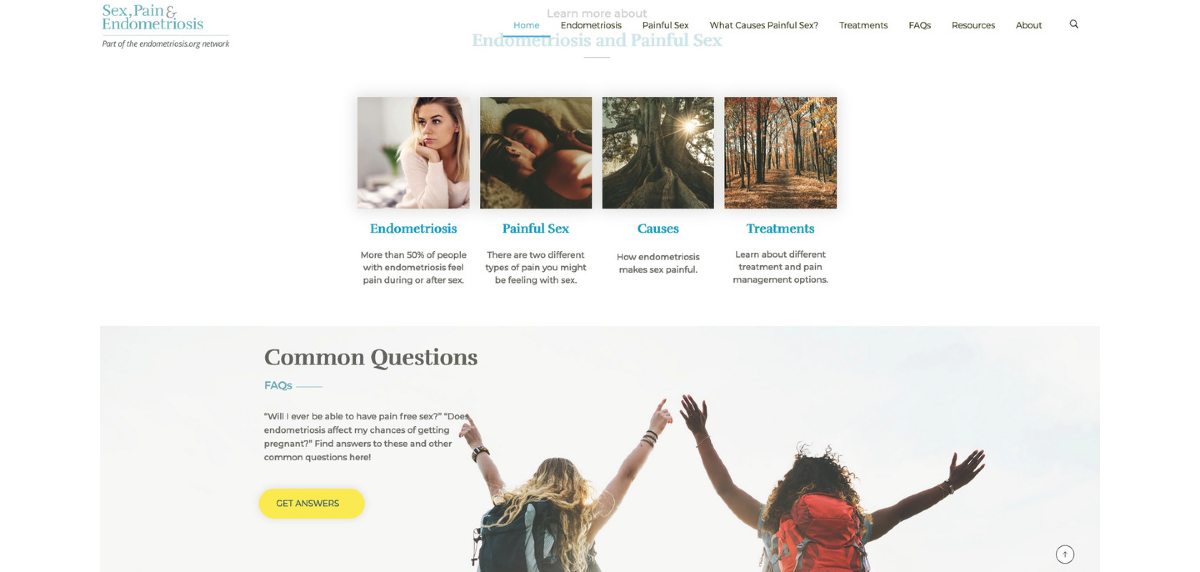
Other sections of the website.

The website was developed by a multidisciplinary team of scientists, health care professionals, patient partners, and community organizations in close collaboration with a web design company as part of an end-of-grant knowledge translation project. We adopted a patient-oriented research approach throughout the website development process wherein patient partners were equal team members in recognition that patients provide critical experience-based perspectives that are essential for creating a meaningful website [13]. The research group drew on the Knowledge to Action Framework and technology-enabled knowledge translation for developing the website [14,15]. We first conducted a needs assessment with our patient partners to determine the scope of the website, content, esthetics, and key messages. Key findings of the needs assessment included prioritizing necessary information on the causes of endometriosis-associated sexual pain and options for treatment. Our patient partners also expressed their desire to develop a website that would promote inclusiveness (eg, diverse gender identities, sexual orientations, ethnocultural backgrounds, and ages) and help address the stigma of endometriosis-associated sexual pain. Although the idea of addressing stigma was not systematically thought through at the time of developing the website, the development team was aware of the need for the website to address stigma in one way or another. Therefore, the images and language used on the website were carefully designed so as not to offend or stereotype website users. We also conducted a landscape analysis of pre-existing endometriosis and sexual pain websites to determine their content and features. This was followed by an iterative product development. Following the release of version 1 of the website, we conducted the usability analysis reported in this paper to determine its usability and functionality and whether the website met the users’ needs. The website was modified based on the findings of the usability analysis before the final launch in February 2021. The main purpose of this website is to help people understand endometriosis-associated dyspareunia, feel empowered, and dispel the myths and misconceptions surrounding endometriosis and sexual pain [9]. The website has six main sections: providing information on endometriosis, painful sex, causes of painful sex, and treatment for painful sex; resources; and frequently asked questions. The process of website development is described in a forthcoming publication. Figure 1 shows the homepage with the main sections of the website and Figure 2 shows infographics of people affected by endometriosis-associated sexual pains and other sections of the website.

The purpose of this study is 2-fold; it aimed to (1) evaluate the usability of the website and (2) assess for destigmatizing properties of sexual health–related web-based resources. The usability evaluation is expected to help improve the web design to make it easy to use, satisfying, and acceptable to end users. We assessed for destigmatizing properties as websites on sensitive health topics are not typically assessed for their ability to address or exacerbate stigma despite stigma being an outcome of interest for content developers [16-18]. That is, it is unclear how the design of digital platforms for general sensitive health problems could help address stigma or inadvertently reproduce and perpetuate stigma among users [19,20].

## Methods

### Overview

We conducted a usability analysis by using think-aloud observations followed by post–think-aloud interviews and a postinterview questionnaire with 12 study participants. Using our website as a reference point, we also asked the participants how sexual health–related websites may be designed to address stigma. Owing to the COVID-19 pandemic, all activities were carried out on the web.

### Recruitment

There have been no studies to our knowledge that provide population-based information on women with endometriosis and dyspareunia in western Canada. However, at the time of this study, there were approximately 300 patients diagnosed with endometriosis who were registered in the Endometriosis Pelvic Pain Interdisciplinary Cohort Data Registry of a large urban health care center in western Canada and who consented to be contacted for future research. Using a systematic sampling approach, we selected every 11th person from a list of approximately 300 patients in the data registry. A total of 45 participants were subsequently contacted. Participants who expressed interest but did not meet the inclusion criteria were excluded. The inclusion criteria were (1) patients aged ≥18 years; (2) new patients or patients rereferred to the center between May 1, 2019, and December 31, 2019; (3) consent to be contacted for future research; (4) patients known to have clinically suspected or diagnosed endometriosis; and (5) experience of self-reported deep sexual pain (alone or partnered). The exclusion criteria were (1) not sexually active (alone or partnered), (2) never experienced sexual pain (alone or partnered), and (3) not fluent in English.

### Ethics Approval

Ethics approval was obtained from the Children’s and Women’s Research Ethics Board at the University of British Columbia (research ethics board approval number: H19-03556). Of the 45 participants contacted, 12 (27%) consented to take part in the study.

### Data Collection

#### Demographics

Before other data collection, the participants were asked to complete a demographic questionnaire that included age, gender identity, ethnicity, computer skills, and the frequency of computer use.

#### Usability Analysis

We conducted a usability analysis by using think-aloud observations, a Post-Study System Usability Questionnaire (PSSUQ), and a follow-up interview. In the think-aloud procedure, we asked the participants to carry out 5 task scenarios on our website while sharing their screens. The task scenarios represented different ways of searching for information about endometriosis-associated dyspareunia. These included (1) the meaning of *cul-de-sac* in relation to endometriosis, (2) different ways of treating sexual pain, (3) information on fertility, (4) anticipation of the pain cycle, and (5) the role of nervous system sensitization in painful sex. The participants were allowed to begin with any of the tasks. The think-aloud observations were conducted remotely via Zoom (Zoom Video Communications, Inc), all sessions were audio recorded, and the shared screen was video recorded. The recording did not include the participants’ faces to protect their privacy. The PSSUQ was conducted to evaluate the overall system usefulness, information quality, and interface quality [21]. Adapted from Stinson et al [22], we conducted a follow-up interview to understand aspects of usability that were not made obvious during the think-aloud procedure.

#### Stigma Analysis

Given that some sexual health–related technologies may inadvertently exacerbate stigma [19,20], the follow-up interviews also included questions to understand the ways in which such websites can be designed to address stigma. In other words, the focus of the interviews was to use our website as a reference point to identify generalities about how destigmatizing sexual health–related websites could be designed. The interviews were audio recorded and transcribed verbatim by the first author (AFA).

### Data Analysis

#### Usability Analysis

The think-aloud video data were analyzed by the first author based on the coding scheme by Kushniruk and Patel [23] for analyzing think-aloud data in patient information systems. Using the coding scheme as a guide, the first author watched and annotated the videotape and audio recordings with usability problems. Usability problems in the video and audio data were categorized into the five main thematic areas from the study by Kushniruk and Patel [23]: (1) navigation problems (related to the user finding desired information, icons, and labels), (2) comprehensiveness of graphics and text (problems related to the participants’ understanding of labels, icons, and content), (3) system responses (how the website responded to the users’ actions), (4) information content (which aspects of the system contained too much or too little information), and (5) mismatch between the website and the users’ expectations. As the analysis proceeded, additional labels that were not captured in the initial coding scheme but emerged during the analysis were added. A second person (GP) independently reviewed all the video recordings, marked the usability problems, and categorized them based on the 5 thematic areas. The 2 reviewers’ results were compared, and the differences were resolved by discussion. Content analysis procedures were used to identify additional usability problems in the follow-up interviews, whereas demographics and the PSSUQ were analyzed with descriptive statistics (1=minimum and 7=maximum, where lower scores indicate better satisfaction and higher scores indicate poor satisfaction). Problems identified from the follow-up interviews were also categorized under the 5 themes in the framework by Kushniruk and Patel [23].

#### Stigma Analysis

Data relating to potentially destigmatizing properties of sexual health–related websites were analyzed using a deductive approach to qualitative content analysis [24]. The deductive analysis followed a trauma-informed care framework [25] to identify the destigmatizing properties of websites. Trauma and stigma are inherently intertwined [26]. As such, we suggest that a trauma-informed care framework is relevant for analyzing data elicited from participants about stigma as it may inform recommendations to address stigma concerns among people who use sexual health–related websites [25,26]. The 5 principles of trauma-informed care by Fallot and Harris [25] were used to guide the analytic approach. These principles are (1) emotional safety (ensuring services are welcoming), (2) choice (ensuring individuals have options over their treatment and life), (3) collaboration (sharing power and making decisions with individuals), (4) trustworthiness (providing clear, credible, and consistent information about the condition), and (5) empowerment (providing an atmosphere that allows individuals to feel validated and affirmed). All transcripts were uploaded into NVivo software (version 11; QSR International). Using the steps for conducting deductive thematic content analysis by Braun and Clark [24], the first author initially familiarized himself with the data. Second, codes and concepts related to each of the 5 principles of the trauma-informed care framework were assigned to the text. Third, patterns and themes were searched for across the different interviews. Finally, the codes and concepts together with the subthemes were matched with their respective global themes, also known as the principles of trauma-informed care. This deductive approach allowed for the systematic identification of the participants’ perspectives across 3 levels, including global themes (ie, the 5 principles of trauma-informed care), subthemes, and concepts. Data saturation was achieved when no new concepts were identified in the data. The coding was discussed with coauthors FH and LC until a consensus was achieved.

## Results

### Demographic Information

The participants’ ages ranged from 30 to 63 years, with a mean age of 38.75 (SD 8.55) years. All participants (12/12, 100%) self-identified as heterosexual, and all were from a large metropolitan area in western Canada. A total of 11 participants (11/12, 92%) self-identified as women, and 1 participant (1/12, 8%) did not disclose. Of the 12 participants, 6 (50%) identified as White, 2 (17%) identified as Hispanic, 1 (8%) identified as Indigenous, and 3 (25%) did not disclose their ethnic identity. All participants (12/12, 100%) reported using the internet daily, 42% (5/12) described their computer skills as very good, 50% (6/12) indicated that their computer skills were quite good, and 8% (1/12) indicated that their skills were neither good nor bad.

### Task Completion

Tasks that required finding information in the *Mechanisms* section were the most difficult for the participants to complete, followed by finding the meaning of *cul-de-sac* in the *Endometriosis* section (Table 1). All participants (12/12, 100%) were able to locate information in the *Treatment* section, and 92% (11/12) of the participants located the information on fertility. Tasks were considered incomplete if the participants were assisted by the researcher (AF) or if they made several mistakes before locating the item. Table 1 shows the task completion rate and the average time (in seconds) it took the participants to complete each task. The entire study took approximately 1.2 hours for each participant to complete.

**Table 1:** Task completion rate (N=12).

Task number	Task	Website section	Participants completing the task, n (%)	Time (seconds), mean (SD)
1	Find the meaning of *cul-de-sac* in relation to endometriosis	Endometriosis	9 (75)	165 (23)
2	Find the different ways of treating sexual pain	Treatment	12 (100)	50 (15)
3	Find the information on fertility	FAQs^a^	11 (92)	80 (12)
4	Locate the anticipation of pain cycle	Mechanisms	8 (67)	206 (19)
5	Find the role of nervous system sensitization in painful sex	Mechanisms	7 (58)	260 (26)

^a^FAQs: frequently asked questions.

### Overall Usability

Most participants expressed minimal difficulty with the think-aloud procedure, although a few had to be occasionally reminded to speak out loud. All participants (12/12, 100%) were able to complete the tasks, although 3 participants (3/12, 25%) needed some hints such as *please click on this link* or *the tab is located up there*. Generally, the participants were happy with the layout of the website. Participant 3, a woman aged 63 years, said the following:

This is a very simple website...I am not bombarded with too much information, the writing is good for my age...I don’t have to strain my eyes to read this.

### Usability Problems

The think-aloud observations and the postsession interviews produced 30 usability problems, of which 23 (77%) were identified via the think-aloud process, and 7 (23%) were identified in the postsession interviews. Table 2 contains the problems identified from the usability analysis organized according to the 5 thematic areas by Kushniruk and Patel [23], the location of each problem on the website, the number of times the problem occurred, and the number of users encountering the problem. The problems were largely content-related issues, particularly related to a preponderance of provider perspectives on the website. For instance, it was generally agreed that the content of the website was medically oriented and currently lacked patients’ perspectives on endometriosis and sexual pain. The participants also suggested solutions to some of the usability problems they identified (Table 3). The mean overall PSSUQ score was 2.41 (SD 0.85) in the range of 1 to 7, indicating relatively high satisfaction. Although the participants’ scores across the 3 PSSUQ metrics were all below the average PSSUQ scores, system usefulness had the lowest score, indicating a better metric of usability. This was followed by interface quality and information quality. Table 4 shows the mean score for each PSSUQ item as well as the overall mean score for system usefulness, information quality, and interface quality.

**Table 2:** Interface problems from usability analysis.

Category and number			Usability problem	Page location	Times occurred, n	Users encountering the problem, n (%)
**Navigation problems**						
	1	There is no search bar.		Home	15	12 (100)
	2	The information underneath the homepage is not apparent to users.		Home	8	5 (42)
	3	Links to other websites open on the same page. Difficult to navigate back to main page.		Home	6	3 (25)
	4	The *go to top* icons at the bottom of the pages are not immediately visible.		Several	5	2 (17)
	5	The sexual response cycle diagram does not fit in the screen for a whole view.		Mechanism	6	3 (25)
	6	In-text references are not directly linked to the reference list. Users have to scroll up and down in search of references.		Several	5	5 (42)
	7	Treatment pop-ups are too small. Not convenient to users.		Treatment	9	6 (50)
	8	Not enough hyperlinks and hypertext to redirect users to different but related pages.		Several	7	5 (42)
**Comprehension of graphics and text**						
	9	*Mechanism* section not clearly understood.		Home	14	12 (100)
	10	The *slider* affordance on the diagram showing signs of endometriosis portrays a click function rather than a slider.		Endometriosis	6	8 (67)
	11	Users did not understand the term *dyspareunia.*		Pain types	5	8 (67)
	12	Not enough affordances to prompt users to click on diagrams and text.		Several	8	6 (50)
	13	Too much content in treatment options and *mechanisms*.		Treatment	4	3 (25)
**System response**						
	14	The link to *entry pain* does not respond.		Pain types	9	7 (58)
	15	Pages load quite slowly. Takes an average of 8-10 seconds.		Several	8	7 (58)
	16	Meaning on labels not immediately apparent to users.		Several	5	4 (33)
**Information content**						
	17	Too much text in treatment pop-up.		Treatment	6	3 (25)
	18	Endometriosis is not explained on the homepage.		Home	3	3 (25)
	19	Not enough content on symptoms except the description of sexual pain.		Symptoms	2	2 (17)
	20	Content on treatment pop-ups is too cluttered.		Treatment	5	4 (33)
	21	Patient perspectives or voices are lacking on the website. It is medically oriented.		Several	3	4 (33)
**Mismatch between the system and users’ expectations**						
	22	Images are too cheerful to portray feelings of pain.		Symptoms	4	5 (42)
	23	Some bolded text looked like hypertext but was not responsive when users clicked on it.		Mechanisms	4	4 (33)
	24	Clicking on *psychological aspects of sexual pain* takes the user to the *Symptoms* page.		Treatment	3	3 (25)
	25	Clicking on *learn more about how the nervous system and low arousal contribute to painful sex* takes the user to the *Symptoms* page.		Treatment	12	11 (92)
	26	Clicking on *pain types* takes the user to the *Symptoms* page.		Pain types	5	4 (33)
	27	Users think *anticipation of pain cycle* is located in *pain types*.		Home	2	2 (17)
	28	The information underneath each section is not apparent until the section is opened.		Home	6	4 (33)
	29	Links to treatment options are currently limited to only the image and not the entire box where the image is located.		Treatment	11	9 (75)
	30	The website is 1-sided in favor of female partners.		Several	5	5 (42)

**Table 3:** Suggested design solutions.

Number	Problem	Suggested solution
6	In-text references are not directly linked to the reference list. Users have to scroll up and down in search of references.	Clicking on a reference should take the user directly to that reference.
7	Treatment pop-ups are too small. Not convenient to users.	Pop-ups should open on a new page.
13	Too much content in treatment options and mechanisms.	Bullet points are preferred.
16	Meaning on labels not immediately apparent to users.	Automatically display label meanings when hovering around the image.
20	Content on treatment pop-ups is too cluttered.	Bullet points are preferred.
21	Patient perspectives or voices are lacking on the website. It is medically oriented.	Include psychosocial aspect of sexual pain.
28	The information underneath each section is not apparent until the section is opened.	A drop-down menu under each section is preferred.
29	Links to treatment options are currently limited to only the image and not the entire box where the image is located.	Extend the link to the entire box.
30	The website is 1-sided in favor of female partners.	Include male images.

**Table 4:** Post-Study System Usability Questionnaire.

Category and item		Score^a^, mean (SD)	
**System usefulness**			
	Overall, I am satisfied with how easy it is to use this website.		2.44 (0.93)
	It was simple to use this website.		2.00 (0.78)
	I was able to complete the task and scenarios quickly using this website.		2.38 (1.02)
	I felt comfortable using this website.		1.88 (0.51)
	It was easy to learn to use this website.		1.88 (0.66)
	I believe I can know about sexual pain quickly using this website.		2.25(1.05)
	Mean overall score		2.13 (0.68)
**Information quality**			
	The website gave me error messages that told me that something went wrong.		4.63 (1.23)
	Whenever I made a mistake using the system, I could recover easily and quickly.		3.00 (1.01)
	The information provided on the website is clear.		2.13 (0.98)
	It is easy to find the information I need.		2.25 (0.67)
	The information is effective in helping me complete the tasks and scenarios.		2.38 (0.94)
	The organization of the information on the screen is clear.		2.13 (0.77)
	Mean overall score		2.36 (0.78)
**Interface quality**			
	The user interface of this website was pleasant.		1.75 (0.48)
	I would like to use this website.		2.25 (0.73
	This website has all the functions and capabilities I expect it to have.		2.75 (1.03)
	Overall, I am satisfied with this website.		2.38 (0.78)
	Mean overall score		2.28 (0.56)

^a^Lower scores indicate better metrics of usability.

### Destigmatizing Properties of Sexual Health–Related Websites

#### Overview

**Figure 3:**
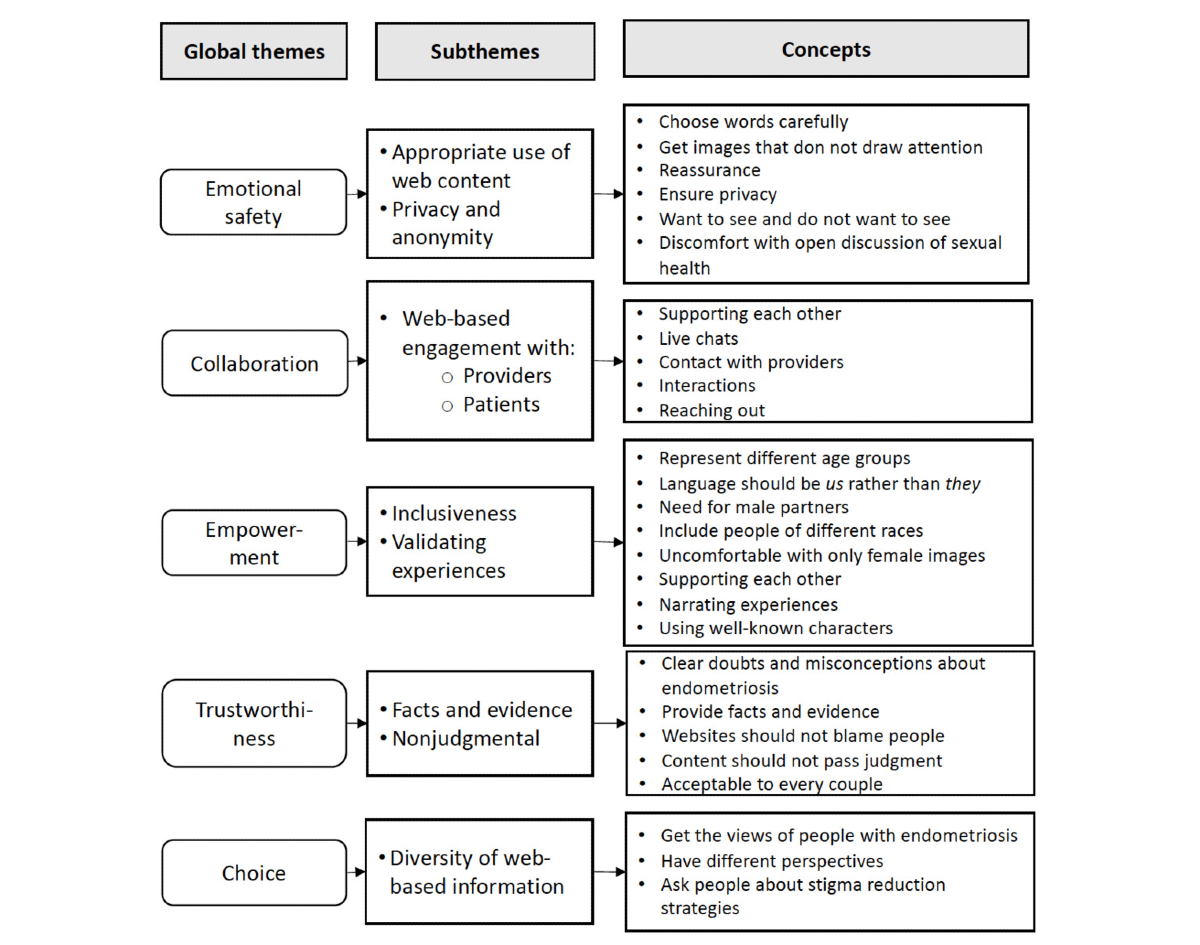
Global themes, subthemes, and concepts based on trauma-informed care.

Although the focus of the stigma analysis was to identify generalities about designing destigmatizing sexual health–related websites, the participants largely referred to our website to illustrate their points. We identified participant responses that fit within the 5 main principles of the trauma-informed care framework by Fallot and Harris [25]. The 5 principles of trauma-informed care represent the global themes under which various subthemes and concepts emerged. These themes represent the participants’ perspectives on how to design destigmatizing sexual health–related websites. Figure 3 shows the data analysis structure based on the trauma-informed care framework.

#### Theme 1: Emotional Safety

##### Overview

According to Fallot and Harris [25], the principle of emotional safety denotes that both the setting and interaction within services are psychologically harmless, comfortable, and easy to use. In other words, emotional safety means having an awareness of individuals’ discomfort or unease in using services. On the basis of this conceptualization of emotional safety, most participants in our study viewed websites as platforms that can promote emotional safety by ensuring that users are not restigmatized or retraumatized when using sexual health–related websites. In total, 2 subthemes emerged describing how stigma may be addressed through emotional safety.

##### Appropriate Use of Web Content

An area of concern among the participants related to how images and content are displayed on web platforms. Many of the participants indicated that the display of sexual health–related content, particularly images, determines whether they will use a web platform. Others argued that the use of websites with explicit images will likely draw people’s attention to what the participants are looking at, a phenomenon that many participants disliked. Participant 3, a woman aged 45 years, stated the following:

When I heard this was a sexual pain website, I thought I was going to see some horrible stuff...you know what I mean...but that’s not the case. You have chosen your words carefully and I wouldn’t shy away from browsing this website, even on a bus.

Owing to the potential for some web images to foment stigma, a participant suggested using images or words that would not easily draw people’s attention, especially in public places.

##### Privacy and Anonymity

Another strategy the participants identified as a means to ensure emotional safety via sexual health–related websites was to safeguard the privacy and anonymity of people who use web-based platforms. Several participants desired privacy and anonymity, indicating that they might feel uncomfortable disclosing their information to health care providers or peers. Participant 1, a woman aged 37 years, reported the following:

I would not want to discuss with my doctor or family members because of the stigma. I mean...you know how people behave when it comes to sexual health issues...So I don’t think I will feel comfortable discussing some of the issues you have on this website with my care provider, but I think this website can provide me with what I want without exposing myself.

The participants generally agreed that educational websites such as ours could enable people to access needed information without necessarily disclosing their private information or their identity. Participant 10, a woman aged 51 years, specifically described the following:

Websites should be able to protect peoples’ identity by using anonymous identities during an online interaction with others or health providers.

#### Theme 2: Collaboration

##### Overview

Collaboration denotes partnerships and the recognition that healing happens in relationships and in a meaningful sharing of power, problems, and experiences [25]. Using our website as a reference, several participants indicated that web platforms can provide a novel opportunity for collaboration among people without revealing their identity. This form of collaboration can be either with people with similar conditions or with health care professionals. Collaboration was regarded as important because many participants indicated that people with sexual pain tend to experience pain alone, in isolation, and with limited or no opportunities to discuss or share their experiences with others. A main subtheme emerged under collaboration.

##### Web-Based Engagement

The desire to interact with peers via websites was a dominant theme described by most participants. For instance, participants 2 and 8 suggested the use of web-based chat rooms to promote active engagement among people with similar problems or to engage with health care providers for additional support. In reference to our website, participant 2 specifically stated the following:

Maybe find a way of connecting people, like, have a place where people would want to hear from each other.

More than half of the participants suggested that contact information for health professionals could also be integrated into sexual health–related websites so that people could always establish an anonymous connection with health care providers for additional information that may not be contained on web platforms. This was specifically echoed by participant 11, a woman aged 39 years:

If you have the contacts of people or those behind the website where I can contact them in case I need personalized information would be useful...don’t you think this will enhance the credibility and relevance of the information?

Although several participants generally agreed on the potential benefit of some form of web-based engagement, a few others were worried about the potential privacy risk of web-based engagement. A participant contended that a person’s identity could inadvertently be revealed through such engagements.

#### Theme 3: Empowerment

##### Overview

In the context of trauma-informed care, empowerment is the recognition that an individual’s strengths are acknowledged, built on, and validated [25]. In other words, empowerment is aimed at conveying a sense of optimism and hope or expanding resources or an individual’s capabilities. When asked how websites may help address stigma, several participants reported strategies that reflected the principle of empowerment by Fallot and Harris [25]. These participants described empowering actions that could promote a sense of optimism and hope among people who use sexual health–related websites. These empowering strategies were categorized under the 2 main subthemes of inclusiveness and validating personal experiences.

##### Inclusiveness

Almost all the participants noted that inclusiveness of people from diverse ethnicities, age groups, and sexes and genders on sexual health–related web platforms is an important strategy to address sexual health–related stigma. For instance, there was consensus across several participants that the presence of partners in this type of web platform was crucial in promoting emotional well-being and addressing stigma-related concerns. Approximately 6 (50%) of the participants suggested including images of partners as well as having a resource section on what partners can do to support and empower their partners who experience sexual pain. Citing our website as an example, participant 4, a woman aged 33 years, voiced the following:

I definitely think you need to get...partners involved in this as well because I think that’s quite important...you [are] definitely not gonna fix this problem or make things easier if one party is trying to do this in isolation without some perspectives from the other party or why they can’t understand what the other party is experiencing...so I think you definitely need to get that involvement in there.

##### Validating Personal Experiences

Several participants noted validating each other’s experiences as an empowering strategy that may help address stigma. Participant 6, for instance, expressed the desire for a section on websites where participants can narrate their experiences as a way of empowering other people who have similar conditions. With specific reference to our website, she noted the following:

I think having a section where I can read about other people’s stories or hear someone narrate their experiences may be helpful to me.

Similarly, participant 4 indicated the following:

...having personal experiences on websites can help people who are living in isolation because such experiences can help them overcome stigmatizing problems that they otherwise would not want other people to know about.

Validating other people’s experiences was considered by many as a way to make people feel understood when using web-based platforms. A few participants also felt that having their experiences validated by others would mean they were not experiencing it in isolation.

#### Theme 4: Trustworthiness

##### Overview

Trustworthiness denotes clarity in tasks and information, accuracy, consistency, transparency, and interpersonal boundaries [25]. Applying this principle in the context of health technologies, many participants reported strategies that they thought could help dispel the myths and misconceptions regarding the causes and possible effects of endometriosis and sexual pain. For instance, participant 3 indicated that the “mere existence of a website that accurately explains endometriosis and sexual pain could help dispel the myths and misconceptions that often fuel stigma.” Participant 6, a woman aged 39 years, also indicated the following:

A website such as this will clear a lot of doubts that many people are having regarding endometriosis and how sexual pain comes about.

The facts and evidence on our website were also seen as ways of promoting trust in web-based content. The use of factual content and nonjudgmental information were the 2 subthemes that emerged under the global theme of trustworthiness.

##### Factual Content

Several participants viewed factual content as a way of addressing stigma via websites. They specifically noted that the information on our website was very factual and based on available evidence, as seen in the references. A participant suggested that other sexual health–related websites should emulate the way our website provided factual content. Participant 6 emphasized the following:

Just as I have said before, the information on all websites should be backed by evidence like you have here. I can see you have references to some of your claims so I can know that there is some credibility to what I am reading.

Although several participants were positive about the facts on the website, a few others viewed the website as too medically oriented. In other words, the website did not portray patients’ perspectives on endometriosis-associated sexual pain. For instance, participant 2 noted that “you only presented the facts without highlighting the psychosocial aspects or patient experiences of sexual pain.”

##### Nonjudgmental Information

Several participants commented on and appreciated the nonjudgmental nature of the information on the sexual health–related website. With reference to our website, participant 2, a woman aged 32 years, reported the following:

Your message does not seem to cast doubts or pass judgment on people who suffer from endometriosis so it can be trusted by people.

The nonjudgmental nature of the information was seen by participants as key to addressing the stigma that results from othering and stereotyping of people who live with an illness. Participant 4 indicated the following:

The information is not personal in nature so I don’t think it may be stigmatizing. I don’t think it also stereotypes anyone.

#### Theme 5: Choice

##### Overview

Choice is the recognition of an individualized approach while strengthening people’s experiences of options in services [25]. In this study, approximately 6 (50%) of the participants indicated ways in which a website might provide diverse information that maximizes an individual’s choices or the diversity of options from which they can choose. A subtheme that emerged under choice related to the diversity of treatment information and opinions on web-based platforms.

##### Diversity of Web-Based Information

Approximately 6 (50%) of the participants indicated that, for a website to address stigma, it should facilitate people’s choices by including diverse information on the treatments, supports, and resources for endometriosis used by a range of people. Awareness of diverse approaches to treating and managing endometriosis and sexual pain was considered by many to be essential to understanding the various approaches and options they might seek further information about or even consider. Considering the private nature of endometriosis and painful sex, there were currently few opportunities to learn from others about what they had tried, both successfully and unsuccessfully. Participant 7, a woman aged 42 years, specifically saw a benefit in asking people who have endometriosis about how they manage stigma related to sexual pain. She said that “asking these people about stigma will help you develop something that can address the problem.” Participant 5 indicated that having different perspectives on the website will maximize people’s choice of sexual pain and stigma management strategies:

Maybe my final suggestion will be to also get different views of people suffering from sexual pain into the website. I think it’s good to hear from different perspectives of how people manage the problem.

## Discussion

### Principal Findings

This study aimed to assess the usability of the *Sex, Pain, and Endometriosis* website and assess for destigmatizing properties of sexual health–related websites in general. The usability findings revealed that, except for the absence of a search bar, the possible confusion with the *mechanisms* tab, and the small pop-up windows, the participants generally found the website to be simple, uncluttered, quite easy to use, and satisfying. The system usefulness, information quality, and interface quality scores on the subscales of the PSSUQ were <3 on average, indicating good usability and satisfaction with the website. It is possible that the system usefulness, information quality, and interface quality were generally perceived as positive as the information was presented in plain language and the website was quite basic so as not to pose usability challenges. In addition to the good PSSUQ ratings, during the posttest interviews, the participants also perceived the content of the website to be credible, evidence-based, nonjudgmental, and appropriate for the age group most affected by endometriosis-associated dyspareunia. The use of nonjudgmental and age-appropriate content on the website confirms the findings of previous studies [27]. The positive findings in this study are in sharp contrast to a review that found the content of 54 endometriosis websites to be fairly inaccurate, of poor quality, noncredible, and fairly difficult to read [10]. The participants also provided suggestions and recommendations, including improving the visibility of the homepage information, explaining endometriosis and dyspareunia on the home page, and providing a drop-down menu under each section heading to make it easier to find content. Despite the positive findings, the largely minor usability problems encountered suggested the need for some revisions and redesign before the website was launched. The participants were unsatisfied with the use of text-heavy information on the website. These findings also remind us that, although more text may be needed to explain certain concepts, the frequent use of dense text on educational websites may not be favorable. Alternatively, images and bullet points could be more engaging to the participants. The recommendations from the research participants informed the revision of the website in ways that better met the needs of potential users. This evaluation study indicated the importance of user feedback in designing patient-centered educational websites on sensitive and intimate health topics [28].

To the best of our knowledge, this work is the first usability evaluation to assess for destigmatizing properties of sexual health–related web-based resources. Even though the participants found the website to be generally nonstigmatizing, the findings of the stigma analysis suggest that the website did not fully use the necessary stigma-alleviating strategies that can empower people to address stigma. For instance, the absence of male partners and inadequate information on patients’ personal experiences were seen as nonempowering. The limitations of our website in addressing stigma could be attributed to the fact that we did not systematically incorporate stigma prevention into our design process even though it emerged during our needs assessment and landscape analysis. Despite these (minimal) setbacks, this study provides preliminary evidence that suggests a trauma-informed approach may inform strategies that can help web designers in developing destigmatizing websites. This is important as web platforms are increasingly used to disseminate information on sensitive health topics [11]. However, no strategies or guiding principles exist to help designers address stigma on websites despite stigma being a major issue in sexual health–related topics. As trauma and stigma are inherently intertwined and work to reinforce each other [26,29], these findings suggest that adopting a trauma-informed approach to developing digital platforms may help address stigma concerns among users of web-based resources. For instance, the anonymous web-based channels identified under the principle of collaboration may motivate people to reach out when in need of support, provide avenues for web-based consultation or engagement with health professionals, and provide a channel for sharing coping mechanisms and supporting each other to overcome the stigma of pain symptoms. The findings of this study demonstrate how other studies that have adopted a trauma-informed approach to successfully design interventions have helped address stigma concerns among people living with HIV and AIDS in other settings [29].

This study also shows how inclusiveness can be applied in web design to address sexual health–related stigma. Inclusive design approaches suggest all-encompassing ways in which websites could be optimized to be usable and acceptable to diverse populations with respect to ethnicity, gender, age, and other forms of human differences. Although inclusiveness was a concern among the patient partners during our needs assessment, we cannot say that our website fully maximized the principles of inclusiveness as the participants noted the absence of male partners during the usability analysis. Future websites on sensitive health topics should start with the principles of inclusive design from the outset. With the principle of inclusiveness, this study extends the emphasis on inclusive design principles from a predominant focus on older adults and people with disabilities to include diverse human variabilities such as ethnicity, gender, and diversity in perspectives [30].

Although the various strategies identified by the participants could help in creating destigmatizing websites, some of the strategies may have an inherent privacy risk. In other words, some of the strategies may reveal rather than conceal a user’s identity to others. For instance, some participants were worried about the privacy risk of web-based communication features such as chat rooms. This worry was not surprising, as the use of chat rooms and other two-way web-based communication features has been associated with privacy breaches in other studies [31]. The participants were not particularly worried about our website as it does not offer any web-based two-way communication features. However, they expressed privacy concerns for other sexual health–related websites that collect personal information. These findings reflect studies that suggest that anonymous websites or websites that do not collect or store any personal information, including ours, may be useful for obtaining information on stigmatized conditions or symptoms such as sexual pain [32,33]. However, the participants’ suggestions for anonymous chat rooms on websites may be an avenue for abusive and web-based stigmatization by peers [16]. This can negate the importance of this potentially safe space. The potential privacy and security risks inherent in chat rooms suggest the need for special design considerations to make anonymous chat rooms or live chats safer.

Given that people may be exposed to all kinds of information from varying sources, the facts and evidence-based information on our website were seen as credible and reliable information that will ultimately address the misconceptions of endometriosis and sexual pain. Although there is still room for improvement, our website also ensures a full range of choices by showing different treatment options for endometriosis-associated dyspareunia. What was missing to fully maximize the principle of choice was having diverse patient perspectives captured on the platform. Our team grappled with providing information about self-management or treatment strategies for which there is only anecdotal evidence, deciding in the end to highlight the information that has greater theoretical or empirical evidence to support its use. Furthermore, the desires for diverse information and for factual information (under the principle of trustworthiness) were very interesting but contradictory to each other, though consistent with our team’s internal debate while creating the website. Although the diversity of web-based information was considered essential to understanding the various options and approaches that people with endometriosis-associated sexual pain might take, evidence may not be available to support all the different options people may desire.

The need for websites to convey real-life experiences and facilitate interaction among people as a way of addressing sexual health–related stigma is consistent with previous studies [17,34]. These previous studies demonstrated how the use of positive language and patients’ personal experiences can promote the uptake of web-based resources. These findings also show that our website may be particularly useful for people who need evidence-based information on endometriosis-associated dyspareunia but are fearful of disclosing or discussing their symptoms with providers.

### Limitations

Instead of in-person usability testing, we opted for remote usability testing to comply with public health orders during the COVID-19 pandemic. We turned off the video to protect the users’ privacy and, therefore, could not observe body gestures and facial expressions that typically convey participants’ reactions during a think-aloud procedure [35]. However, given the sensitive nature of the topic, remote usability testing might have turned out to be a blessing in disguise as the participants had the opportunity to explore the website from their home and in relative anonymity. Although the participants emphasized the important role of partners in managing sexual health–related stigma, our sample included only people diagnosed with endometriosis. This limitation is consistent with the broad sexual health literature, where partner engagement in women’s sexual health research is particularly challenging and, in some cases, disregarded [36-38]. Future usability studies on websites that address sexual health problems should include partners to ensure an inclusive end product. For an educational website on endometriosis-associated dyspareunia such as this one, partner perspectives are particularly important as the psychosocial impact of sexual pain can also be experienced by partners [39].

### Conclusions

Websites on sensitive health topics are increasingly designed to provide anonymous platforms for people to obtain evidence-based information and to empower users, dispel myths, and alleviate stigma.

## Abbreviations

PSSUQ: Post-Study System Usability Questionnaire
